# Duration of Acute Kidney Injury and In-Hospital Mortality in Elder Patients with Severe COVID-19: A Retrospective Cohort Study

**DOI:** 10.1155/2022/9929038

**Published:** 2022-07-26

**Authors:** Yue Cai, Chong Tang, Yun Song, Qinglin Li, Shanshan Guo, Yanyan Chen, Fang Wang, Yangping Li, Yan Lei, Fei Li, Ling Tao

**Affiliations:** ^1^Department of Cardiology, Xijing Hospital, Changle West Road, Xi'an 710032, China; ^2^Huo Shen Shan Hospital, Wuhan, China; ^3^Department of Respiratory Medicine, Xijing Hospital, Changle West Road, Xi'an 710032, China; ^4^Department of Critical Care Medicine, The First Medical Centre, Chinese PLA General Hospital, 28 Fuxing Road, Beijing 100853, China; ^5^Department of Nephrology, Xijing Hospital, Changle West Road, Xi'an, 710032, China

## Abstract

**Background:**

Patients with severe coronavirus disease 2019 (COVID-19) who develop acute kidney injury (AKI) in the intensive care unit (ICU) have extremely high rates of mortality. This study evaluated the prognostic impact of AKI duration on in-hospital mortality in elder patients.

**Methods:**

We performed a retrospective study of 126 patients with confirmed COVID-19 with severe or critical disease who treated in the ICU from February 4, 2020, to April 16, 2020. AKI was defined according to the Kidney Disease Improving Global Outcomes serum creatinine (Scr) criteria. AKI patients were divided into transient AKI and persistent AKI groups based on whether Scr level returned to baseline within 48 h post-AKI.

**Results:**

In total, 107 patients were included in the final analysis. The mean age was 70 (64–78) years, and 69 (64.5%) patients were men. AKI occurred in 48 (44.9%) during their ICU stay. Of these, 11 (22.9%) had transient AKI, and 37 (77.9%) had persistent AKI. In-hospital mortality was 18.6% (*n* = 11) for patients without AKI, 72.7% (*n* = 8) for patients with transient AKI, and 86.5% (*n* = 32) for patients with persistent AKI (*P* < 0.001). Kaplan–Meier curve analysis revealed that patients with both transient AKI and persistent AKI had significantly higher death rates than those without AKI (log-rank *P* < 0.001). Multivariate Cox regression analysis revealed that transient and persistent AKI were an important risk factor for in-hospital mortality in older patients with severe COVID-19 even after adjustment for variables (*hazard* *ratio* [*HR*] = 2.582; 95% CI: 1.025–6.505; *P* = 0.044; and *HR* = 6.974; 95% CI: 3.334–14.588; *P* < 0.001).

**Conclusions:**

AKI duration can be an important predictive parameter in elder patients suffering from COVID-19 and are admitted to ICU. Among these patients, those exhibiting persistent AKI have a lower in-hospital survival rate than those with transient AKI, emphasizing the importance of identifying an appropriate treatment window for early intervention.

## 1. Introduction

Since December 2019, an outbreak of coronavirus disease 2019 (COVID-19) has rapidly evolved into a global pandemic [1]. The COVID-19 epidemic carries an especially higher risk to older populations resulting in severe complications, intensive care unit (ICU) admissions, and high mortality rate [2–5]. On May 2, 2022, 513,523,166 confirmed cases of COVID-19 including 6,261,385 deaths were reported by the World Health Organization.

AKI is frequently observed in COVID-19 infected patients, approximately 26–80% of AKI occurrence in ICU settings [6–9]. AKI has been recognized as a surrogate marker of the severity of illness; the in-hospital mortality is as high as 50% overall [7]. The recommendation from the Kidney Disease Improving Global Outcomes (KDIGO) guidelines defined AKI and classified the stages of AKI severity into three grades, based on the increase and/or decrease in serum creatinine (Scr) and urine output, and a more advanced AKI stage has been associated with adverse outcomes [10, 11]. Irrespective of its severity, AKI duration recently has been viewed as another independent risk factor for a poorer outcome: Longer duration of AKI (also called persistent AKI), typically defined as more than 48–72 h after onset, has been associated with a higher risk for death compared to short-duration AKI (also called transient AKI) [12–17].

Limited information is available on the association between the duration of the increase in Scr and clinical outcomes in severe COVID-19 patients in the ICU [18]. We hypothesized that the majority of AKI would be persistent after AKI development and could be associated with higher short-term mortality. The aim of the current study was to evaluate the incidence and prognostic impact of AKI duration and AKI stage on in-hospital mortality in elder patients with severe COVID-19.

## 2. Patients and Methods

### 2.1. Study Design and Patients

We retrospectively analyzed patients diagnosed with COVID-19 who were hospitalized from February 4, 2020, to April 16, 2020. All patients who were enrolled in this study were diagnosed with COVID-19 according to the guidance provided by the Chinese National Health Commission. This study was approved by the National Health Commission of China and the Institutional Review Board at Huo Shen Shan Hospital (HSSLL028, Wuhan, China). The requirement for written informed consent was waived by the ethics committee of the designated hospital for patients with emerging infectious diseases.

**Figure 1:**
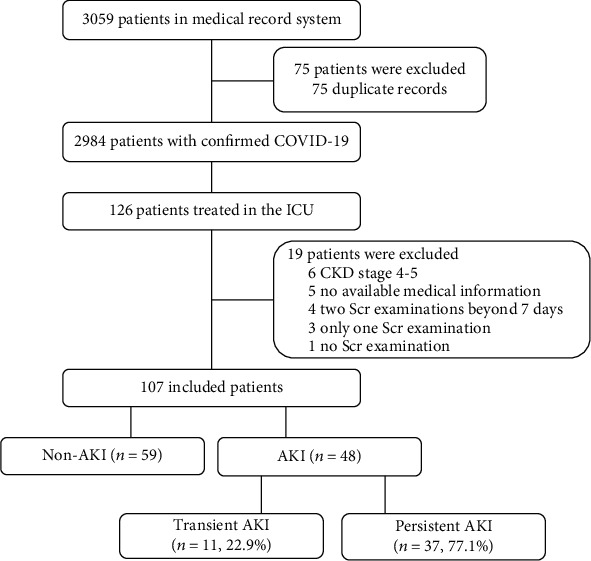


Between February 4, 2020, and April 16, 2020, we followed 126 patients with confirmed COVID-19 with severe or critical disease who required admission to the ICU. Of the 126 patients, we excluded 19 patients, resulting in 107 elderly patients who were eligible for the final analyses, among which 48 (44.9%) had AKI during their ICU stay (Figure 1). We excluded patients who had stages 4–5 CKD, had only 1 Scr test or no Scr examination, or had missing or incomplete medical history. Patients who had 2 Scr assays with intervals longer than 7 days were also excluded because we could not determine whether these patients developed AKI.

### 2.2. Data Collection

Epidemiological, medical records, nursing records, radiological characteristics, treatments, and outcome data were obtained with data collection forms from electronic medical records and reviewed by a trained team of physicians. The information recorded included demographic data, medical history, exposure history, underlying comorbidities, time of symptoms onset, signs, laboratory findings, chest computed tomography (CT) scans, complications (acute respiratory distress syndrome [ARDS], sepsis, septic shock, AKI, hypoproteinemia, and disseminated intravascular coagulation), treatments during hospitalization, and hospitalization and discharge or death. All medical records of patients with AKI were checked by 2 trained intensive care specialists and nephrologists. All patients data accessed complied with relevant data protection and privacy regulations.

### 2.3. Definitions

The criteria in the KDIGO guidelines for Scr levels were used for screening patients because retrospectively collected urine data can be inaccurate [11]. AKI was defined as an increase at least 26.5 *μ*mol/L in Scr within 48 h or a 50% increase from the baseline value within 7 days. “Transient AKI” was defined as Scr that returned to baseline within 48 h post-AKI; “persistent AKI” was defined as renal dysfunction without recovery within 48 h [19]. The baseline Scr level was defined as the most recent measurement in the previous 3 months [20]. When there were no prior records on Scr level, we used the lowest Scr value during hospitalization as the baseline Scr level [21, 22]. ARDS was defined according to the Berlin definition [23]. Septic shock was defined according to the Sepsis-3 criteria [24].

### 2.4. Statistical Analysis

Continuous parametric variables are presented as the means ± standard deviations (SDs), and continuous nonparametric variables are presented as medians with interquartile ranges (25th and 75th percentiles). Categorical variables are presented as numbers (*n*) or percentages (%). Three-group comparisons were conducted using one-way ANOVA or the Kruskal–Wallis H test for continuous variables and Pearson's chi-square test or Fisher's exact test for categorical variables. The associations between AKI and in-hospital death were examined using Cox proportional hazard regression analysis. The probability of survival was estimated using the Kaplan–Meier method, and curves were compared using the log-rank test. All tests were two-sided, and *P* < 0.05 was considered to indicate statistical significance. Statistical analyses were performed using SPSS version 21.0 for Windows (SPSS, Inc., Chicago, IL).

## 3. Results

### 3.1. Development of AKI in Study Population

Figure 1 shows the flow diagram of this study. Of these 48 AKI patients, transient AKI was documented in 11 (22.9%) and persistent AKI in 37 (77.1%) according to KDIGO guidelines. In addition, 55 patients required invasive mechanical ventilation (51.4%), 38 patients required vasopressors (35.5%), and 20 patients required continuous renal replacement therapy (CRRT, 18.7%). During follow-up, a total of 51 patients (47.7%) died, including 11 patients in non-AKI group, 8 in the transient AKI group, and 32 in the persistent AKI group.

### 3.2. Clinical Characteristics Associated with Transient AKI and Persistent AKI

As shown in Table 1 and Table 2, the persistent AKI group had a higher percentage of cardiovascular disease (45.9% vs 36.4% vs 20.3%) or CKD (13.5% vs 0 vs 0) compared with the non-AKI group and transient AKI group. Similarly, the persistent AKI group was more frequently suffered from ARDS (64.9% vs 45.5% vs 32.2%), septic shock (67.6% vs 36.4% vs 15.3%), and more frequently required CRRT (40.5% vs 18.2% vs 5.1%). Laboratory results, such as baseline Scr levels (*P* < 0.001), Scr on ICU admission levels (*P* < 0.001), Scr at the time of AKI diagnosis levels (*P* < 0.001), peak Scr levels (*P* < 0.001), BUN levels (*P* < 0.001), uric acid levels (*P* < 0.001), calcium levels (*P* = 0.038), cystatin C levels (*P* < 0.001), platelets levels (*P* < 0.001), lactate levels (*P* = 0.001), and oxygenation index levels (*P* < 0.011) on ICU admission differed significantly between the three groups. Patients with transient AKI had a higher percentage of cerebrovascular disease (*P* = 0.006); needed for vasopressors (*P* < 0.001); were more frequently treated with mechanical ventilation (*P* < 0.001); and more frequently suffered from oliguria (*P* < 0.001), proteinuria (*P* < 0.001), and hematuria (*P* = 0.023). Patients with persistent AKI more frequently exhibited stages 3 and 2 AKI than patients with transient AKI (48.6% vs 36.4%, 21.6% vs 9.1%; *P* < 0.001).

### 3.3. Acute Kidney Injury and KDIGO Stage

**Figure 2:**
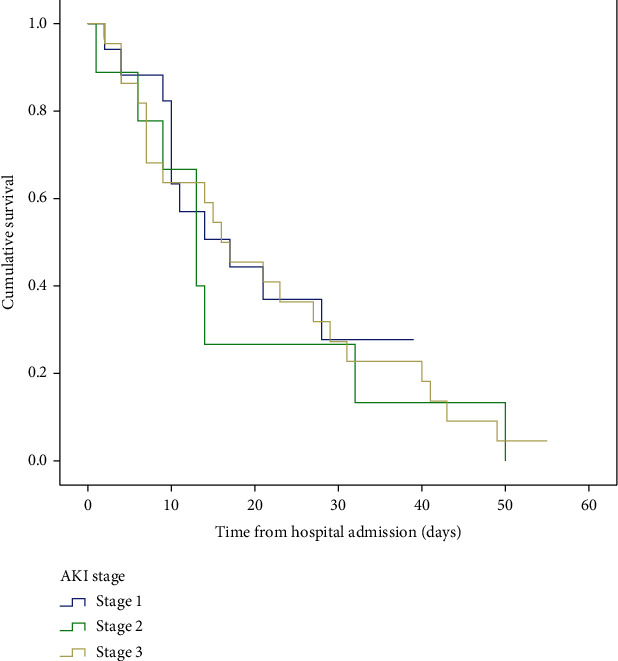


According to the KDIGO criteria, 17 patients (35.4%) had stage 1 AKI, 9 (18.8%) had stage 2 AKI, and 22 (45.8%) had stage 3 AKI. AKI severity was not associated with a significantly higher in-hospital mortality (64.7% for stage 1 patients, 88.9% for stage 2, and 95.5% for stage 3). Surprisingly, outcomes not worsened with more advanced AKI stage (*P* = 0.864 for the three stages, Figure 2).

### 3.4. Effect of Transient and Persistent AKI on In-Hospital Mortality

**Figure 3:**
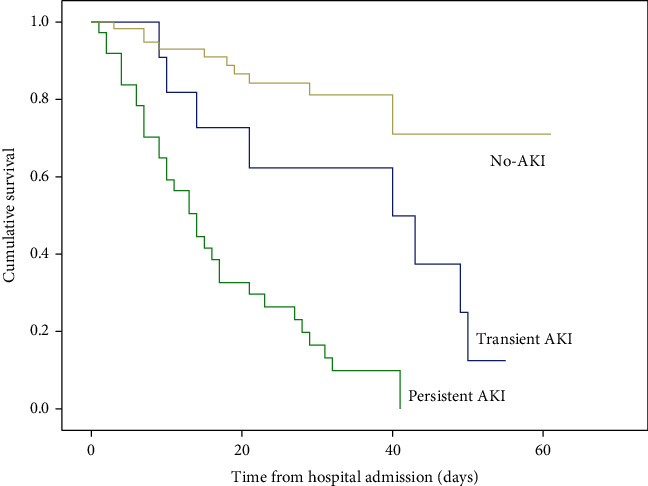


As shown in Table 3, in-hospital mortality was 18.6% (*n* = 11) for patients without AKI, 72.7% (*n* = 8) for patients with transient AKI, and 86.5% (*n* = 32) for patients with persistent AKI (*P* < 0.001). Kaplan–Meier curve analysis revealed that patients with both transient AKI and persistent AKI had significantly higher death rates than those without AKI in elderly patients with severe COVID-19 (log-rank *P* < 0.001; Figure 3). Furthermore, in a multivariable Cox proportional hazard analysis, transient and persistent AKI were an important risk factor for in-hospital mortality in elderly patients with severe COVID-19 even after adjustment for variables (hazard ratio [HR] = 2.582; 95% CI: 1.025–6.505; *P* = 0.044; and HR = 6.974; 95% CI: 3.334–14.588; *P* < 0.001, respectively) (Table 3).

## 4. Discussion

In this observational study, we illustrated that 45% (48/107) of severe patients with COVID-19 developed AKI, including 22.9% (11/48) patients with transient AKI and 77.1% (37/48) patients with persistent AKI. However, these patients with transient AKI showed significantly higher in-hospital mortality than those without AKI. Moreover, we found that patients with persistent AKI had a higher rate of in-hospital mortality than those with transient AKI.

AKI is common among severe patients with COVID-19, affecting approximately 26–76% of patients admitted to the ICU [6–8]. In-hospital mortality rates of AKI range from 50% overall to 42% in the ICU [7]. AKI could be distinguishable in terms of renal recovery from AKI or the duration of AKI [25]. Recent findings have led to the hypothesis that transient AKI and persistent AKI in critically ill patients might share similar pathophysiological mechanisms and that the duration of AKI might reflect its severity rather than its mechanism [26]. In the present study, patients with persistent AKI had significantly higher death rates than those transient AKI. Although exact pathophysiology is not clearly elucidated in, experimental models have shown that even with apparent renal function recovery from AKI, histologic and physiologic changes may persist after AKI. Recently, the 2017 Acute Disease Quality Initiative (ADQI) 16 workgroup defined persistent AKI as the continuance of AKI diagnosed by the Scr level or urine output criteria for >48 hours after AKI onset. So early recognition of transient AKI secondary to severe COVID-19 and use of supportive and therapeutic measures to avoid further kidney damage are crucial to reduce morbidity and mortality. Several studies have examined the prognostic impact of AKI duration using different diagnostic criteria as follows: transient AKI (≤7 days) and persistent AKI (>7 days) using the KDIGO criteria [27, 28]; transient AKI (Scr ≤115 *μ*mol/L at discharge) and persistent AKI (Scr>115 *μ*mol/L at discharge) using the KDIGO criteria [29]; short (≤2 days), medium (3–5 days), and long (≥6 days) using the KDIGO criteria [15]; and transient AKI (≤3 days) and persistent AKI (>3 days) using the AKIN or KDIGO criteria [16, 17]. The use of a universally recognized definition of AKI would improve our knowledge regarding practices for, research on, and public health issues related to AKI.

Previous studies on the prognostic impact of AKI duration yielded conflicting results. For example, Mizota and colleagues published a retrospective cohort study involving 258 AKI patients (median age 66 years) undergoing major abdominal surgery [28]. Using the KDIGO criteria, the postoperative AKI patients were divided into transient AKI and persistent AKI groups based on the time when the Scr level returned to the no-AKI range within 7 days after surgery. The authors reported that both transient AKI and persistent AKI were independently associated with 1-year mortality. Also, the mortality rate was higher in the persistent AKI than the transient AKI group. However, another retrospective study found no significant association between AKI duration and adverse outcomes [17]. The discrepancies between these studies might reflect differences in the duration of recovery from AKI according to the definition of AKI, transient AKI, or persistent AKI; the participants' age; and the etiology of AKI. One of the limitations is that we not further classified persistent AKI into categories based on AKI duration.

In the present study, we found that patients with both transient AKI and persistent AKI had significantly higher death rates than those without AKI in elderly patients with severe COVID-19. The ADQI 16 workgroup defined transient AKI as Scr that returned to baseline within 48 h after AKI onset calling for at least two measurements over a 48-hour period and baseline Scr available prior to admission. However, this can be difficult in clinical practice with emerging infectious diseases. In addition, previous publications from developed countries report the proportion of patients with more than 2 Scr measurements during hospitalization ranging from 63.2 to 67.6%, which is much higher than the figure reported in China only 25% to 30% [30–32]. Therefore, some patients with transient AKI could be misclassified as not having AKI [16]. Indeed, improving survival only via recognized AKI is not enough. It is, for instance, the absence of kidney care that is also a negative factor affecting the outcomes. In 2009, the National Confidential Enquiry into Patient Outcome and Death reported that up to 50% of patients who died from AKI had not received “good” kidney care; in addition, for 20% of these patients, the cause was both predictable and preventable [33]. Thus, increasing the likelihood of a timely AKI diagnosis and identifying patients who need kidney care to preventing the progression of transient AKI to persistent AKI remains a challenge.

The classification of AKI by duration may discriminate between patients with transient AKI (pre-renal) or hemodynamic AKI that does not involve any true injury to the renal tubular cells and those with true intrinsic AKI (that is, structural kidney injury). Furthermore, the duration of AKI may be a surrogate of the renal recovery potential of the injured kidney or continued on-going insults. The parameters used for evaluating patients' renal function were glomerular filtration rate (GFR) or Scr. The recent definition from the KDIGO guideline classifies AKI by increasing severity, from stage 1 to stage 3, based on the Scr increase and/or decrease in urine output, and more severe AKI stages are associated with adverse outcomes [10, 11]. However, we found no association between in-hospital mortality and AKI severity in this study. It has been demonstrated that in elderly patients, renal recovery may be prolonged and frequently incomplete. Additionally, the duration of AKI may likely denote the overall illness severity of the patient, as those who are more severely ill and have continued extrarenal organ dysfunction will take longer to recover. Therefore, the KDIGO diagnosis and staging of AKI with Scr may not be suitable for the elderly population with severe COVID-19. Herein, we demonstrated that the duration of AKI still provided prognostic information over the KDIGO stage alone and can provide additive risk information for mortality risks for elderly patients, especially when AKI duration is longer than 48 hours.

The study had the following limitations. (1) First, medical data of this study were collected from a single center, and the number of patients included is limited; thus, the results may lack generalizability. (2) Second, because of the strain on medical resources, urine output data of most patients were missing and not been collected, which is one component of the AKI definition; therefore, the incidence of AKI may be underestimated. (3) Due to the limited number of patients included, AKI patients were not further divided into categories based on AKI duration, such as short duration: resolving AKI lasting 3–4 days; medium duration: resolving AKI lasting 5–7 days; and long duration: AKI lasting >7 days. Thus, a larger AKI patient population is needed to confirm these results. (4) Finally, we lacked data after the patients were discharged.

## 5. Conclusions

The duration of AKI is independently associated with in-hospital mortality and may provide prognostic information in addition to that provided by the magnitude of Scr alone. Among AKI patients, those persistent AKI have a higher in-hospital survival rate than those transient AKI. These data need to be validated in other settings of AKI, and if found to be valid, the duration of AKI should be incorporated into the consensus definitions of AKI and used in clinical studies of AKI in the future.

## Figures and Tables

**Table 1:** Comparisons of the clinical characteristics of patients between transient AKI, persistent AKI, and non-AKI with coronavirus disease 2019.

Characteristic	Non-AKI group (59, 55.1)	Transient AKI (11, 22.9)	Persistent AKI (37, 77.1)	*P* value ^a^
Age (years)	68 (63–75)	73 (67–81)	73 (66–82)	0.097
Male sex	37 (62.7)	6 (54.5)	26 (70.3)	0.578
Body mass index (kg/m^2^)	22.8 ± 1.9	23.7 ± 1.8	23.7 ± 2.7	0.105
Comorbidity				
Hypertension	37 (62.7)	8 (72.7)	28 (75.7)	0.386
Cardiovascular disease	12 (20.3)	4 (36.4)	17 (45.9)	0.028
COPD	12 (20.3)	1 (9.1)	10 (27.0)	0.388
Diabetes	10 (16.9)	1 (9.1)	11 (29.7)	0.193
Cerebrovascular disease	6 (10.2)	6 (54.5)	7 (18.9)	0.006
CKD	0	0	5 (13.5)	0.004
Signs and symptoms				
Cough	51 (86.4)	7 (63.6)	31 (83.8)	0.234
Fever	46 (78.0)	9 (81.8)	26 (70.3)	0.615
Dyspnea	47 (79.7)	4 (36.4)	26 (70.3)	0.019
Muscle ache	38 (64.4)	5 (45.5)	25 (67.6)	0.413
Fatigue	34 (57.6)	6 (54.5)	21 (56.8)	0.982
Chest pain	11 (18.6)	1 (9.1)	4 (10.8)	0.478
Chills	11 (18.6)	1 (9.1)	4 (10.8)	0.478
Headache	4 (6.8)	1 (9.1)	1 (2.7)	0.582
Diarrhea	1 (1.7)	0	3 (8.1)	0.204
Chest CT findings				
Multiple mottling and ground glass opacity	56 (94.9)	11 (100.0)	33 (89.2)	0.267
Pneumonia				0.549
Unilateral pneumonia	1 (1.7)	0	0	
Bilateral pneumonia	58 (98.3)	11 (100.0)	37 (100.0)	
Disease classification				<0.001
Severe	30 (50.8)	1 (9.1)	6 (16.2)	
Critical	29 (49.2)	10 (90.9)	31 (83.8)	
On ICU admission				
MAP	97 ± 15	94 ± 23	95 ± 21	0.881
Albumin (g/L)	31.9 ± 3.8	30.5 ± 4.4	31.1 ± 4.6	0.493
BUN (mmol/L)	6.1 (4.5–8.1)	7.3 (4.9–9.8)	10.0 (6.4–14.0)	<0.001
Uric acid (*μ*mol/L)	189.0 (133.0–229.0)	225.0 (192.0–378.0)	293.0 (184.0–427.5)	<0.001
Potassium (*μ*mol/l)	4.2 (3.8–4.5)	4.3 (4.1–5.0)	4.2 (3.9–4.8)	0.372
Sodium (mmol/l)	140.0 (137.0–143.0)	138.0 (139.0–144.0)	143.0 (137.0–146.0)	0.095
Chlorine (mmol/l)	103.0 (100.0–106.0)	103.0 (101.0–109.0)	104.0 (102.0–111.0)	0.097
Calcium (mmol/L)	2.0 (1.9–2.2)	2.0 (1.9–2.3)	2.0 (1.9–2.0)	0.038
Phosphate (mmol/L)	0.9 (0.7–1.0)	1.0 (0.7–1.4)	0.9 (0.7–1.1)	0.223
Magnesium (mmol/L)	0.9 (0.8–1.0)	0.9 (0.9–1.0)	1.0 (0.9–1.1)	0.054
Cystatin C (mg/L)	1.0 (0.9–1.2)	1.4 (1.1–1.8)	1.4 (1.1–1.9)	<0.001
Hemoglobin (g/L)	114 ± 20	111 ± 19	122 ± 21	0.135
Platelets (×10^9^/L)	224 (165–276)	189 (65–351)	152 (58–182)	<0.001
C-reactive protein (mg/L)	33.2 (8.7–112.4)	76.0 (11.7–152.6)	92.6 (25.8–151.9)	0.091
Blood gas analysis				
Lactate (mmol/L)	1.2 (0.7–2.3)	1.3 (1.1–3.7)	2.2 (1.5–3.7)	0.001
PH	7.42 ± 0.10	7.40 ± 0.11	7.38 ± 0.13	0.230
PaO_2_ (mmHg)	78.9 (56.7–114.0)	77.3 (61.6–92.0)	71.0 (56.0–89.0)	0.631
PaCO_2_ (mmHg)	38.7 (33.4–45.2)	33.6 (32.0–55.2)	41.0 (35.0–47.0)	0.622
Oxygenation index (mmHg)	205.0 (86.0–280.0)	150.0 (62.0–188.0)	87.0 (66.0–166.0)	0.011
Proteinuria	13 (22.0)	9 (81.8)	24 (64.9)	<0.001
Hematuria	19 (32.2)	7 (63.6)	21 (56.8)	0.023
Kidney function				
Baseline Scr (*μ*mol/L)	58 (48–66)	58 (54–72)	72.0 (65.0–80.5)	<0.001
Scr on ICU admission (*μ*mol/L)	56.8 (48.6–68.8)	78.0 (55.0–95.0)	84.0 (70.0–124.5)	<0.001
Scr at the time of AKI diagnosis (*μ*mol/L)	56.8 (48.6–68.8)	111.4 (95.0–134.2)	131.7 (113.5–164.6)	<0.001
Peak Scr (*μ*mol/L)	67.6 (57.6–77.1)	111.4 (100.0–245.4)	212.5 (136.5–287.5)	<0.001
Oliguria	0	5 (45.5)	12 (32.4)	<0.001
AKI stage				<0.001
1	0	6 (54.5)	11 (29.7)	
2	0	1 (9.1)	8 (21.6)	
3	0	4 (36.4)	18 (48.6)	

Notes: Values are *n* (%), mean ± SD, or median (interquartile range). Abbreviations: AKI: acute kidney injury; COPD: chronic obstructive pulmonary disease; CKD: chronic kidney disease; MAP: mean arterial pressure; 1 mmHg = 0.133 kPa. Scr: serum creatinine; BUN: blood urea nitrogen; ICU: intensive care unit. ^a^*P* values represent the comparability across the three groups.

**Table 2:** Complications, treatments and outcomes of patients between transient AKI, persistent AKI, and non-AKI with coronavirus disease 2019.

Characteristic	Non-AKI group (59, 55.1)	Transient AKI (11, 22.9)	Persistent AKI (37, 77.1)	*P* value^a^
Complications				
Acute respiratory distress syndrome	19 (32.2)	5 (45.5)	24 (64.9)	0.007
Hypoproteinemia	22 (37.3)	4 (36.4)	15 (40.5)	0.941
Septic shock	9 (15.3)	4 (36.4)	25 (67.6)	<0.001
Disseminated intravascular coagulation	3 (5.1)	1 (9.1)	5 (13.5)	0.358
Treatment				
Antibiotic therapy	55 (93.2)	11 (100.0)	35 (94.6)	0.492
Glucocorticoids	51 (86.4)	10 (90.9)	30 (81.1)	0.651
Intravenous immunoglobulin therapy	32 (54.2)	4 (36.4)	25 (67.6)	0.149
Need for vasopressors	9 (15.3)	7 (63.6)	22 (59.5)	<0.001
Oxygen therapy	57 (96.6)	9 (81.8)	33 (89.2)	0.163
Non-invasive mechanical ventilation	26 (44.1)	10 (90.9)	31 (83.8)	<0.001
Invasive mechanical ventilation	16 (27.1)	9 (81.8)	30 (81.1)	<0.001
Continuous renal replacement therapy	3 (5.1)	2 (18.2)	15 (40.5)	<0.001
Extracorporeal membrane oxygenation	1 (1.7)	1 (9.1)	2 (5.4)	0.419
Time from symptom onset to hospital admission (days)	16 (12–26)	14 (7–22)	13 (8–20)	0.035
Time from symptom onset to ICU admission (days)	21 (14–36)	24 (17–33)	17 (14–26)	0.136
Length of hospital stay (days)	26 (16–40)	32 (14–49)	13 (7–23)	<0.001
Length of ICU stay (days)	9 (5–15)	6 (3–36)	7 (4–13)	0.618
In-hospital mortality	11 (18.6)	8 (72.7)	32 (86.5)	<0.001

Abbreviations: AKI: acute kidney injury; ICU: intensive care unit. ^a^*P* values represent the comparability across the three groups.

**Table 3:** Multivariate proportional hazard model analysis of risk factors for hospital mortality.

Risk factor	HR	95% CI	*P* value
Non-AKI	Reference	Reference	<0.001
Transient AKI	2.582	1.025–6.505	0.044
Persistent AKI	6.974	3.334–14.588	<0.001
Oxygenation index	0.992	0.988–0.996	<0.001

Abbreviations: AKI: acute kidney injury; HR: hazard ratio; CI: confidence interval.

## Data Availability

The datasets used and/or analyzed during the current study are available from the corresponding authors on reasonable request.

## Abbreviations

COVID-19: Coronavirus disease 2019
SARS: Severe acute respiratory syndrome
MERS: Middle East respiratory syndrome
ICU: Intensive care unit
ARDS: Acute respiratory distress syndrome
ECMO: Extracorporeal membrane oxygenation
AKI: Acute kidney injury
KDIGO: Kidney disease: improving global outcomes
CRRT: Continuous renal replacement therapy.

## References

[1] Daneshkhah A., Agrawal V., Eshein A., Subramanian H., Roy H. K., Backman V. (2020). Evidence for possible association of vitamin D status with cytokine storm and unregulated inflammation in COVID-19 patients. *Aging Clinical and Experimental Research*.

[2] Grasselli G., Zangrillo A., Zanella A. (2020). Baseline characteristics and outcomes of 1591 patients infected with SARS-CoV-2 admitted to ICUs of the Lombardy Region, Italy. *JAMA*.

[3] Bhatraju P. K., Ghassemieh B. J., Nichols M. (2020). Covid-19 in critically ill patients in the Seattle region - case series. *The New England Journal of Medicine*.

[4] Arentz M., Yim E., Klaff L. (2020). Characteristics and outcomes of 21 critically ill patients with COVID-19 in Washington state. *JAMA*.

[5] Guan W. J., Ni Z. Y., Hu Y. (2020). Clinical characteristics of coronavirus disease 2019 in China. *The New England Journal of Medicine*.

[6] Gabarre P., Dumas G., Dupont T., Darmon M., Azoulay E., Zafrani L. (2020). Acute kidney injury in critically ill patients with COVID-19. *Intensive Care Medicine*.

[7] Chan L., Chaudhary K., Saha A. (2021). AKI in hospitalized patients with COVID-19. *Journal of the American Society of Nephrology*.

[8] Yu Y., Xu D., Fu S. (2020). Patients with COVID-19 in 19 ICUs in Wuhan, China: a cross-sectional study. *Critical Care*.

[9] Rubin S., Orieux A., Prevel R. (2020). Characterization of acute kidney injury in critically ill patients with severe coronavirus disease 2019. *Clinical Kidney Journal*.

[10] Kane-Gill S. L., Sileanu F. E., Murugan R., Trietley G. S., Handler S. M., Kellum J. A. (2015). Risk factors for acute kidney injury in older adults with critical illness: a retrospective cohort study. *American Journal of Kidney Diseases*.

[11] Kellum J. A., Lameire N., Aspelin P. (2012). Kidney disease: improving global outcomes (KDIGO) acute kidney injury work group. KDIGO clinical practice guideline for acute kidney injury. *Kidney international supplements*.

[12] Bellomo R., Ronco C., Mehta R. L. (2017). Acute kidney injury in the ICU: from injury to recovery: reports from the 5th Paris international conference. *Annals of Intensive Care*.

[13] Coca S. G., King J. T., Rosenthal R. A., Perkal M. F., Parikh C. R. (2010). The duration of postoperative acute kidney injury is an additional parameter predicting long-term survival in diabetic veterans. *Kidney International*.

[14] Federspiel C. K., Itenov T. S., Mehta K., Hsu R. K., Bestle M. H., Liu K. D. (2018). Duration of acute kidney injury in critically ill patients. *Annals of Intensive Care*.

[15] Han S. S., Kim S., Ahn S. Y. (2013). Duration of acute kidney injury and mortality in critically ill patients: a retrospective observational study. *BMC Nephrology*.

[16] Li Q., Zhao M., Wang X. (2017). The impact of transient and persistent acute kidney injury on short-term outcomes in very elderly patients. *Clinical Interventions in Aging*.

[17] Perinel S., Vincent F., Lautrette A. (2015). Transient and persistent acute kidney injury and the risk of hospital mortality in critically ill patients: results of a multicenter cohort study. *Critical Care Medicine*.

[18] Mehta S., Chauhan K., Patel A. (2018). The prognostic importance of duration of AKI: a systematic review and meta-analysis. *BMC Nephrology*.

[19] Chawla L. S., Bellomo R., Bihorac A. (2017). Acute kidney disease and renal recovery: consensus report of the acute disease quality initiative (ADQI) 16 workgroup. *Nature Reviews. Nephrology*.

[20] Chao C. T., Tsai H. B., Wu C. Y. (2015). The severity of initial acute kidney injury at admission of geriatric patients significantly correlates with subsequent in-hospital complications. *Scientific Reports*.

[21] Wang Y., Wang J., Su T. (2017). Community-acquired acute kidney injury: a nationwide survey in China. *American Journal of Kidney Diseases*.

[22] Cheng X., Wu B., Liu Y., Mao H., Xing C. (2017). Incidence and diagnosis of acute kidney injury in hospitalized adult patients: a retrospective observational study in a tertiary teaching Hospital in Southeast China. *BMC Nephrology*.

[23] Force A. D., Ranieri V. M., Rubenfeld G. D. (2012). Acute respiratory distress syndrome: the Berlin definition. *JAMA*.

[24] Singer M., Deutschman C. S., Seymour C. W. (2016). The third international consensus definitions for sepsis and septic shock (Sepsis-3). *JAMA*.

[25] Uchino S., Bellomo R., Bagshaw S. M., Goldsmith D. (2010). Transient azotaemia is associated with a high risk of death in hospitalized patients. *Nephrology, Dialysis, Transplantation*.

[26] Schneider A. G., Bellomo R. (2013). Urinalysis and pre-renal acute kidney injury: time to move on. *Critical Care*.

[27] Kim C. S., Bae E. H., Ma S. K., Kweon S. S., Kim S. W. (2016). Impact of transient and persistent acute kidney injury on chronic kidney disease progression and mortality after gastric surgery for gastric cancer. *PLoS One*.

[28] Mizota T., Dong L., Takeda C. (2019). Transient acute kidney injury after major abdominal surgery increases chronic kidney disease risk and 1-year mortality. *Journal of Critical Care*.

[29] Choi J. S., Kim Y. A., Kim M. J. (2013). Relation between transient or persistent acute kidney injury and long-term mortality in patients with myocardial infarction. *The American Journal of Cardiology*.

[30] Yang L., Xing G., Wang L. (2015). Acute kidney injury in China: a cross-sectional survey. *Lancet*.

[31] Xu X., Nie S., Liu Z. (2015). Epidemiology and clinical correlates of AKI in Chinese hospitalized adults. *Clinical Journal of the American Society of Nephrology*.

[32] Lu R., MuciAo-Bermejo M. J., Armignacco P. (2014). Survey of acute kidney injury and related risk factors of mortality in hospitalized patients in a third-level urban hospital of Shanghai. *Blood Purification*.

[33] Macleod A. (2009). NCEPOD report on acute kidney injury-must do better. *The Lancet*.

